# Supplementary research on K150del variant of activated protein C

**DOI:** 10.18632/aging.202904

**Published:** 2021-04-25

**Authors:** Wen-Yi Lin, Liang Tang, Xuan Lu, Yu Hu

**Affiliations:** 1 Institute of Hematology, Union Hospital Affiliated to Tongji Medical College, Huazhong University of Science and Technology, Wuhan, China

**Keywords:** activated protein C, cytoprotective activity, virus defense, 2019 coronavirus disease

## Abstract

Activated protein C (APC) is an anticoagulant with potent cytoprotective and anti-inflammatory effects. K150del, a natural variant of APC, is associated with reduced anticoagulant activity. We performed a comprehensive study to analyze the functional alterations of the K150del mutant. Transcriptome analysis of HEK 293T cells treated with wild and mutant APC revealed differentially expressed genes enriched in inflammatory, apoptotic, and virus defense-related signaling pathways. Both wild and mutant APC displayed concentration-dependent cytoprotective effects. Low concentrations of K150del mutant resulted in decreased anti-inflammatory and anti-apoptotic activities, whereas its higher concentrations restored these effects. Expression of virus defense-related genes improved in mouse lung tissues after repeated administration of the APC variant. These results suggest that the APC K150del mutant could help clinicians to accurately predict disease risks and serve as a potential auxiliary therapeutic in viral infections, including 2019 coronavirus disease (COVID-19).

## INTRODUCTION

Protein C (PC) is a 62 KDa, vitamin K-dependent plasma serine protease that is transformed to its active form, activated protein C (APC) by the thrombomodulin–thrombin complex on the surface of endothelial cells [1, 2]. PC activation is further enhanced by its binding to endothelial protein C receptor (EPCR) [3]. As part of the anticoagulant system, APC, together with various cofactors, such as protein S, factor V (FV), and phosphatidylserine, degrades factors Va and VIIIa and inhibits the production of thrombin [4]. Reduced anticoagulant activity of APC has been implicated in various coagulation disorders. For example, heterozygous deficiency of APC is associated with increased risk of thrombophilia, manifested as recurrent deep venous thrombosis of lower limbs or pulmonary embolism, whereas its homozygous deficiency causes embryonic lethality, developmental anomaly, and fatal thrombotic complications in infants [5]. Furthermore, APC interacts with protease-activated receptor (PAR) 1 and EPCR to exert its cytoprotective functions including beneficial gene alterations, anti-inflammatory and anti-apoptotic activities, and endothelial barrier stabilization [2, 4]. APC has therapeutic applications in several clinical diseases, such as severe sepsis, ischemic stroke (IS), acute respiratory distress syndrome, and tissue repair [6–9]. For instance, recombinant human APC (rhAPC) reduced mortality risk in patients with severe sepsis in the PROWESS trial by decreasing systemic inflammation and controlling the spread of infection [10]. Similarly, the APC variant (3K3A-APC) displayed superior neuroprotection in preclinical models of IS by reducing neurological damage and promoting neurogenesis [11].

K150del is a natural variant of APC with a lysine deletion (AAG) in exon 7 [12]. This variant was first identified in Japanese patients with venous thrombosis and then as a common genetic risk factor for venous thrombosis and IS in the Chinese population [13]. The affected individuals had different thrombotic tendencies, manifested as poor recognition of Arg506 cleavage site of FV due to impaired interaction of APC with protein S. Furthermore, the APC variant was reported to be associated with impaired cytoprotective effects and reduced cleavage of PAR1 and PAR3 [14, 15]. We studied the cytoprotective activity of the K150del variant and defined its molecular mechanisms for clinical phenotypes that could assist the clinicians to evaluate the disease risks of carriers, assess the risk score of patients, and make optimal therapeutic decisions.

## RESULTS

### RNA-seq analysis

**Figure 1:**
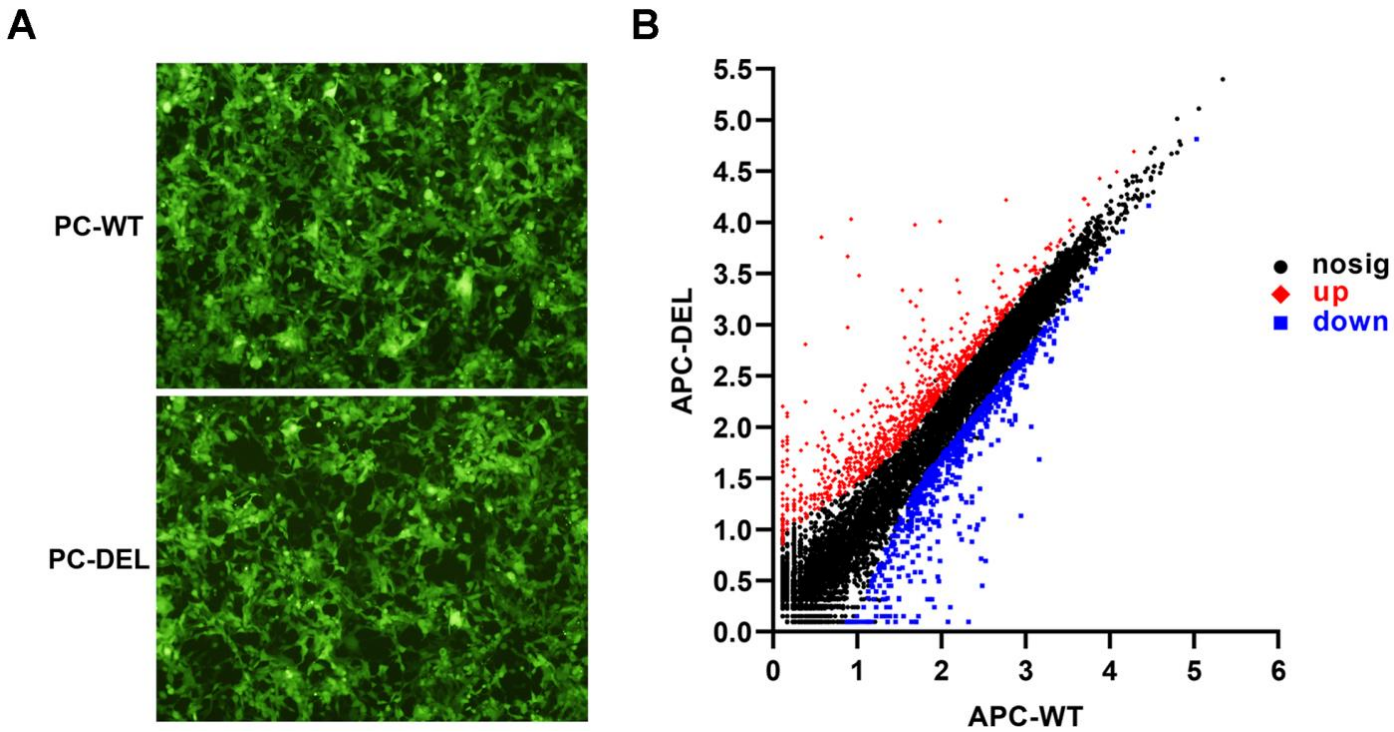
**Establishment and characterization of HEK 293T cell lines.** (**A**) Wild or mutant protein C (PC) zymogen and green fluorescent protein (GFP) were co-expressed in HEK 293T cells using lentiviral vectors. GFP-positive clones were observed under a fluorescence microscope to evaluate the efficacy of two transfections. (**B**) Differentially expressed genes (DEGs) were identified between APC-WT and APC-DEL groups. Each dot represents an individual gene. The horizontal axis indicates the expression (log10) of the genes in the APC-WT group, whereas the vertical axis refers to gene expression (log10) in the APC-DEL group. Red dots represent upregulated genes, blue dots represent downregulated genes, and black dots represent unaltered genes. The significance level for the difference in the expression of each gene between the two groups was set at adjusted *P* < 0.01 and log2FC ≥ 1 or ≤ −1 (FC: gene expression changes).

HEK 293T cells expressing wild or mutant PC zymogen and green fluorescent protein (GFP) were screened. The GFP-positive clones were observed under the microscope as a reference for the efficacy of two transfections (Figure 1A). Transcriptome analysis of HEK 293T cells treated with wild or mutant APC (APC-WT and APC-DEL) revealed genetic alterations in two transformants. A total of 1,484 differentially expressed genes (DEGs) were identified with 831 genes upregulated and 653 genes downregulated in the mutant APC group (adjusted *P* < 0.01 and log2FC ≥ 1 or ≤ −1) (Figure 1B).

**Figure 2:**
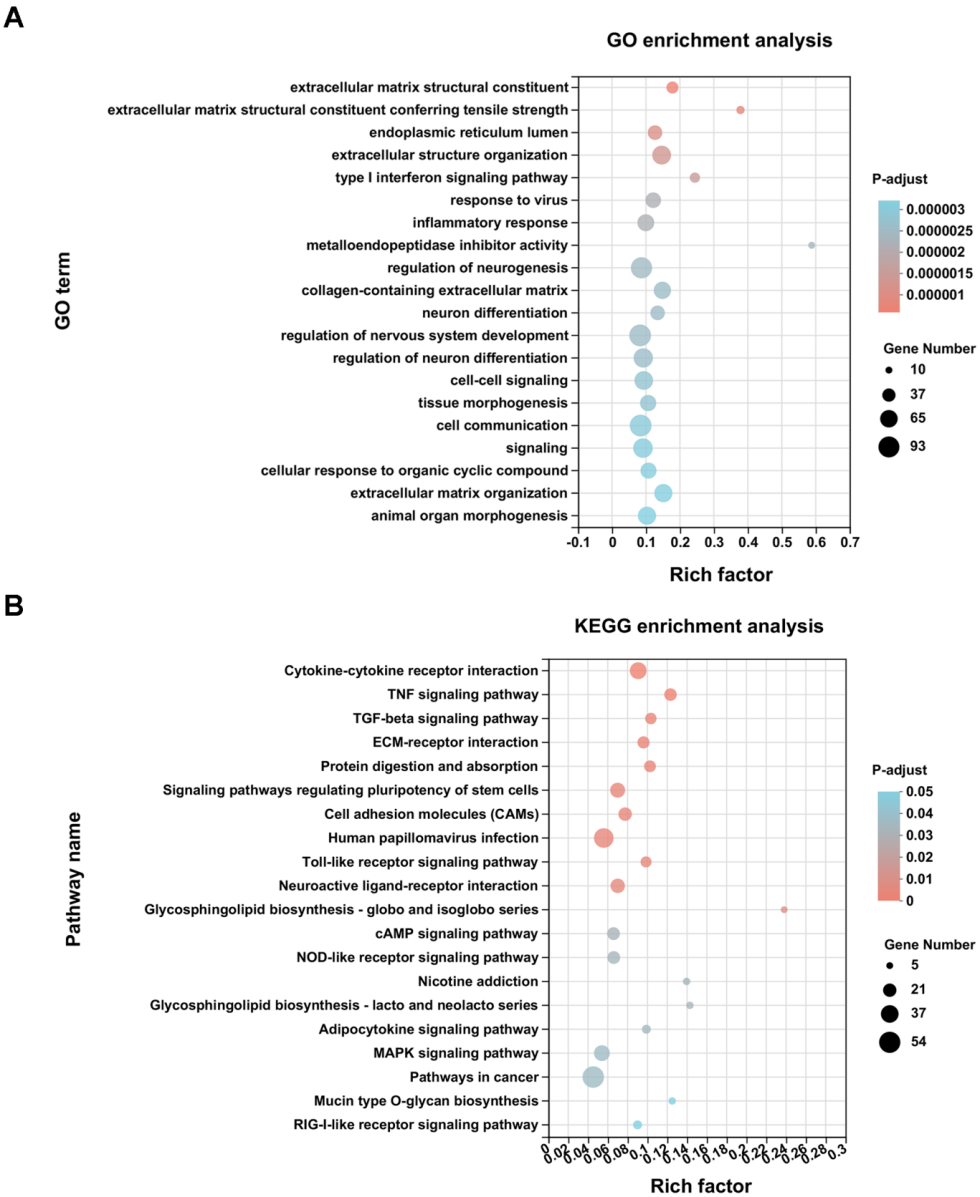
**Enrichment analysis of DEGs.** (**A**) GO enrichment analysis of differentially expressed genes (DEGs) was conducted with the top 20 ranked GO terms and shown as a bubble diagram. The vertical axis indicates GO terms and the horizontal axis represents the rich factor. The higher the rich factor, the stronger the enrichment score. The size of dots indicates the number of genes in the GO term, and the color of dots represents adjusted *P* for different terms. (**B**) KEGG enrichment analysis of DEGs was performed with top 20 ranked KEGG signaling pathways recognized and shown as a bubble diagram.

Figure 2A showed the top 20 Gene Oncology (GO) enrichment analysis terms. Metalloendopeptidase inhibitor activity displayed the strongest enrichment score with the highest rich factor (0.59), followed by an extracellular matrix structural constituent conferring tensile strength (rich factor: 0.38). Extracellular structure organization, tissue morphogenesis, virus-related immune responses, inflammatory response, nervous system development, and cell–cell communications were additional enriched biological processes. The Kyoto Encyclopedia of Genes and Genomes (KEGG) pathway analysis of DEGs revealed 20 signaling pathways (adjusted *P* < 0.05), of which 11 pathways, mainly enriched in biological metabolism, interaction and transduction of signaling molecules, and responses to viral infection, displayed substantial differences (adjusted *P* < 0.01) (Figure 2B).

**Figure 3:**
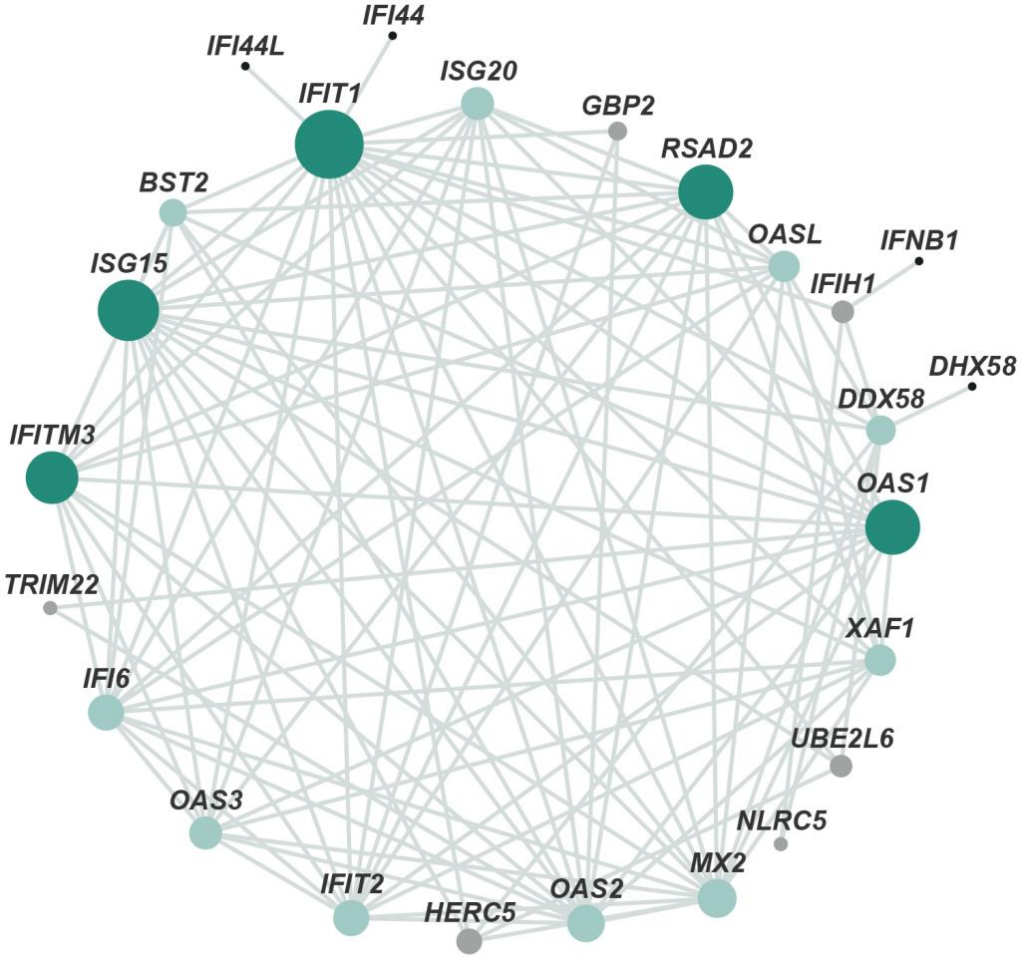
**Protein–protein interaction network of DEGs.** The main network cluster of DEGs was mapped from 205 genes and 300 relationships. Each node represents a protein and each edge represents an interaction between two proteins. Node color from black to green represents lowest to highest betweenness centrality (BC). The size of each node corresponds to the degree (number of connections).

In addition, the identified protein–protein interaction (PPI) networks consisted of 300 relationships (edges) and 205 genes (nodes) (Figure 3). IFIT1, ISG15, RSAD2, OAS1, and IFITM3, factors that regulate the generation of type I interferon (IFN) and immune responses to viruses, such as inhibition of virus proliferation and elimination of virus-infected cells, constituted networks with high degree of centrality. These results, except for virus-defense responses, were consistent with those of previous studies that implicated APC in development [5], anti-inflammatory and anti-apoptotic activities [2, 4], and neuroprotection [11].

### Purification of APC and detection of anticoagulant activity

**Figure 4:**
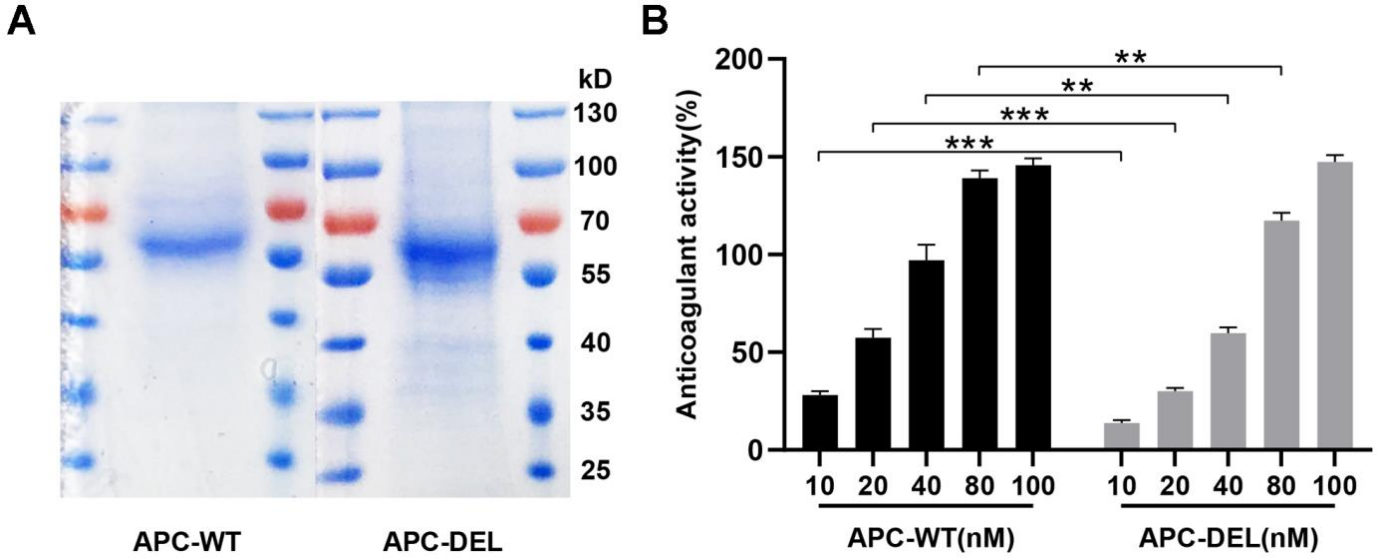
**Characterization of wild and mutant APC.** (**A**) SDS-PAGE and Coomassie staining of APC-WT and APC-DEL protein samples were performed under non-reducing conditions. (**B**) Anticoagulant activities of increasing concentrations of APC-WT and APC-DEL. Differences between wild and mutant APC at the same concentration were evaluated (*P < 0.05, **P < 0.01, ***P < 0.001).

As shown in Figure 4A, APC-WT and APC-DEL were purified to homogeneity. There was a positive correlation between the anticoagulant activity of APC mutant and its concentrations. The anticoagulant activity of APC-DEL was impaired at low concentrations (10 nM, 20 nM, 40 nM, and 80 nM) (49.1 ± 7.7%, 52.4 ± 2.0 %, 61.6 ± 2.8%, and 84.6 ± 1.0% of that of APC-WT). A markedly higher concentration (100 nM) was required to achieve a similar anticoagulant effect to that of the wild type (Figure 4B). These results were consistent with those of previous studies that reported differences in the anticoagulation activity between normal APC and the K150del variant [12, 13].

### Anti-inflammatory activity

**Figure 5:**
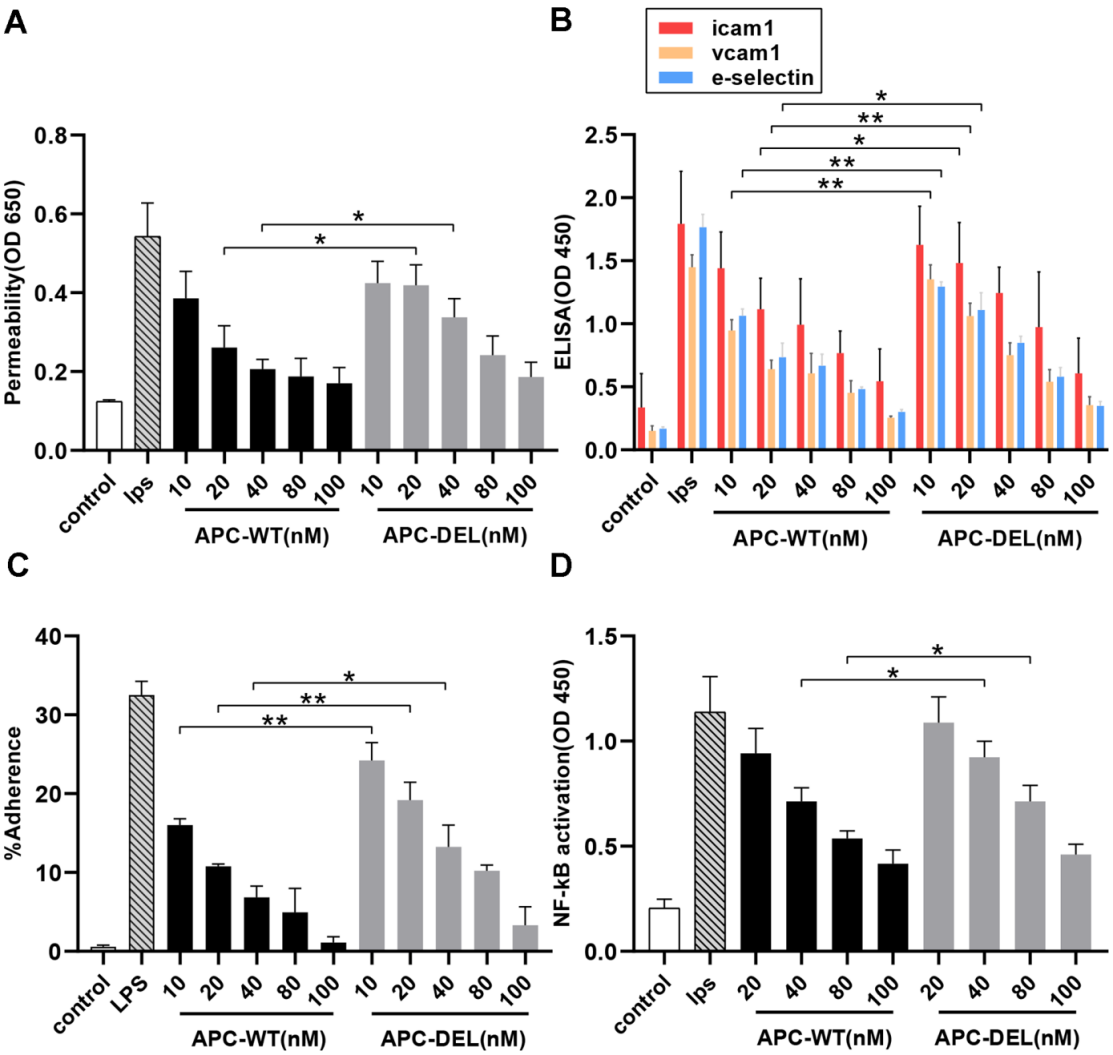
**Anti-inflammatory activity of APC derivatives using cellular assays.** The anti-inflammatory activity of different concentrations of APC derivatives was assessed using four cellular assays as described in the “Methods” section. All results are presented as means ± SD of three different experiments. The significance level, *P < 0.05, **P < 0.01 and ***P < 0.001, represents differences in the APC-DEL group compared to the APC-WT group. (**A**) Protective effects of APC-WT and APC-DEL on the endothelial cell permeability in response to LPS. The permeability was determined spectrophotometrically at 650 nm. (**B**) Effects of APC-WT and APC-DEL on LPS-induced expression of cell adhesion molecules, ICAM-1, VCAM-1, and E-selectin, on the surface of endothelial cells. The expression was measured using an ELISA kit and detected at 450 nm. (**C**) The inhibitory effect of APC-WT and APC-DEL on the adherence of THP-1 cells to LPS-activated endothelial cells. The percentage of adherent THP-1 cells was calculated using the following formula: % adherence = (adherent signal/total signal) x 100. (**D**) The inhibitory effect of APC-WT and APC-DEL on LPS-induced NF-κB activation. The degree of activation of NF-κB was measured using an ELISA kit and detected at 450 nm.

APC protects the endothelial cells from lipopolysaccharide (LPS)-induced hyperpermeability effect [4, 16]. Both APC-WT and APC-DEL exhibited protective effects and reduced the barrier permeability of vascular endothelial cells in a concentration-dependent manner (Figure 5A). Although 20 nM APC-WT inhibited LPS-induced barrier permeability, a higher concentration (40 nM) of APC-DEL was required to achieve a similar permeability-inhibiting efficacy. At further higher concentrations (80 nM and 100 nM), APC-DEL displayed a cytoprotective effect similar to that of APC-WT, reflected by the elimination of the barrier disruptive effect of LPS.

We next evaluated the effect of APC on the expression of cell adhesion molecules, namely intercellular adhesion molecule-1 (ICAM-1), vascular cell adhesion molecule-1 (VCAM-1), and E-selectin on the surface of endothelial cells. Both APC-WT (10 nM) and APC-DEL (40 nM) inhibited the proinflammatory function of LPS and the cell surface expression of these adhesion molecules. At 40 nM and higher concentrations, APC-DEL exhibited inhibiting capacities similar to those of APC-WT (Figure 5B). The reduced expression of adhesion molecules correlated with the decreased binding of THP-1 cells to LPS-activated endothelial cells. Compared with APC-WT, the inhibitory effect of APC-DEL on leukocyte infiltration was less, about 50.9 ± 8.8%, 61.7 ± 7.1%, and 74.4 ± 8.9% of that of APC-WT (at 10 nM, 20 nM, and 40 nM, respectively). Compared with the LPS-free group, both APC-WT and APC-DEL reduced the adhesion of THP-1 cells at higher concentrations (Figure 5C).

LPS upregulates the inflammatory pathways by activating NF-κB in endothelial cells [4, 16]. As shown in Figure 5D, APC-WT and APC-DEL effectively inhibited the activation of NF-κB in endothelial cells. Furthermore, the inhibitory capacity of APC-DEL against NF-κB activation was weaker (about 49.0 ± 10.3% and 70.6 ± 3.8% at 40 nM and 80 nM, respectively) than that of APC-WT. At 100 nM, APC-DEL displayed a protective effect against LPS-induced NF-κB activation similar to that of APC-WT.

### Anti-apoptotic activity

**Figure 6:**
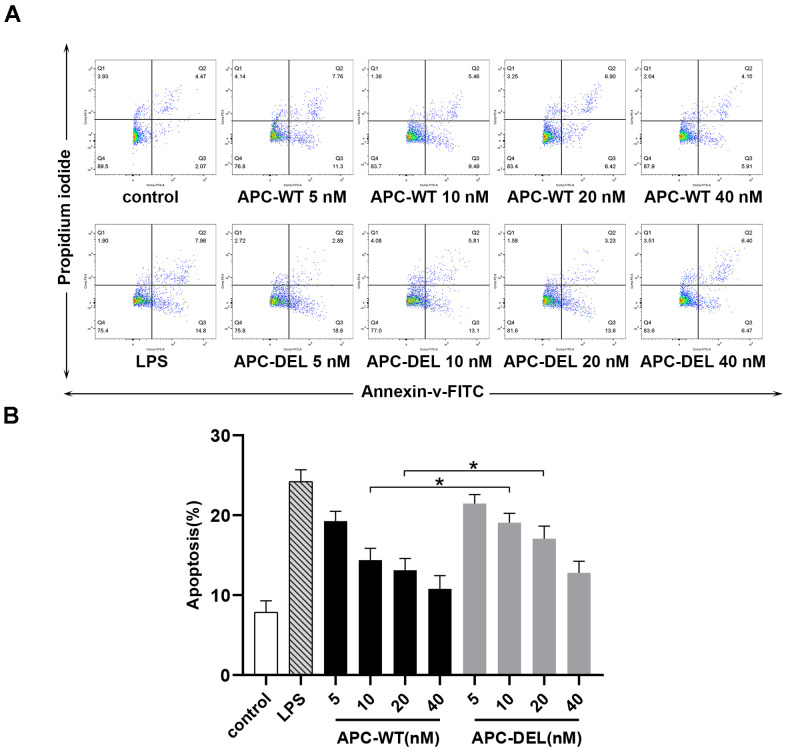
**Cytoprotective effects of APC derivatives in response to staurosporine-induced apoptosis.** (**A**) The anti-apoptotic activity of APC-WT and APC-DEL in HUVECs in response to staurosporine was evaluated using the Annexin V–FITC apoptosis detection kit. The everted phosphatidylserine in cell membranes, probed by Annexin V labeled with FITC, reflected early apoptosis (Q2), whereas the exposed cell nuclei, probed by propidium iodide, reflected middle and late apoptosis (Q3). The total apoptosis rate was equal to Q2 plus Q3. (**B**) Statistical analysis of data derived from three independent measurements shown in (**A**).

Staurosporine is known to induce apoptosis in endothelial cells [17]. As shown in Figure 6, staurosporine increased the apoptosis of endothelial cells at a rate about thrice that observed in the untreated group. Both APC-WT and APC-DEL displayed cytoprotective effects in endothelial cells in a concentration-dependent manner. A concentration of 5 nM of APC-WT decreased staurosporine-induced apoptosis. However, the anti-apoptotic activity of APC-DEL was less, requiring a higher concentration (10 nM) to achieve inhibitory effects on apoptosis in endothelial cells. At 40 nM, APC-DEL exhibited protective responses similar to those of APC-WT against staurosporine-induced apoptosis.

### Anti-viral effects

**Figure 7:**
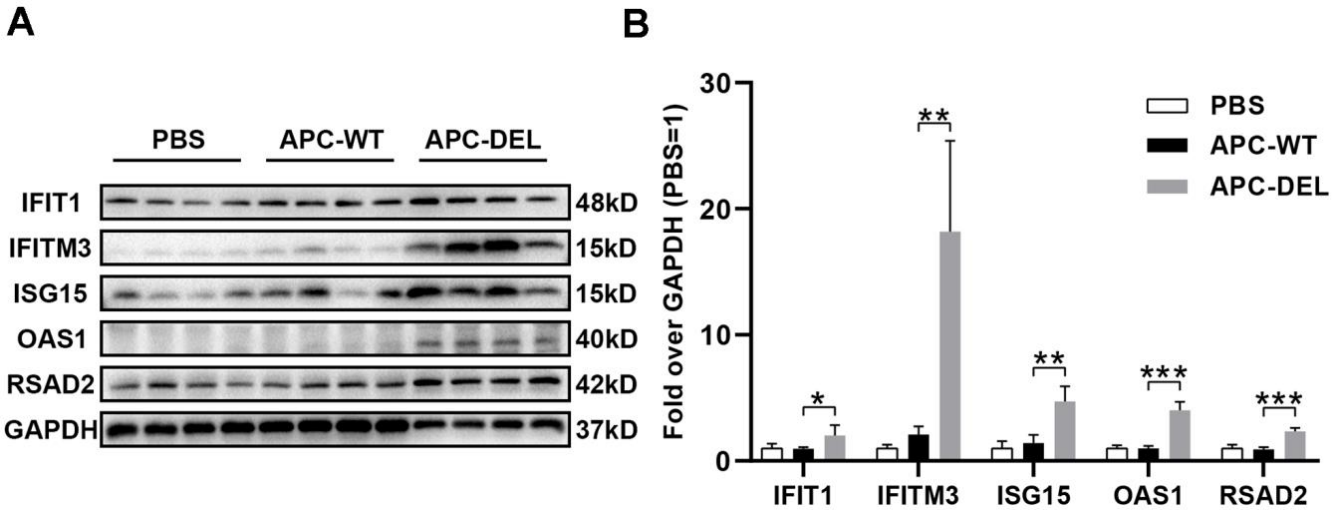
**Anti-viral effects of APC derivatives.** (**A**) Western blot analysis of IFIT1, IFITM3, ISG15, OAS1 and RSAD2 antigens in mice lung tissues (*n* = 4 for each group) after multiple injections with PBS, APC-WT, or APC-DEL. GAPDH antigen was used as the internal control. (**B**) The intensity value of each band was analyzed using the Image Lab software. The significance level was set at *P < 0.05, **P < 0.01 and ***P < 0.001 (compared with the APC-WT group).

Transcriptome analysis revealed that APC-DEL activated the pathways involved in IFN generation and immune responses against viral infections. To determine the anti-viral effects of APC-DEL *in vivo*, mice were administered high doses of APC-DEL. As shown in Figure 7A, the expression of IFIT1, IFITM3, ISG15, OAS1, and RSAD2 in mice lung tissues was markedly increased following frequent treatments with APC-DEL; the increase was much higher than that in APC-WT or PBS-treated group. Among these factors, IFITM3 was substantially affected, the expression of which was about 6 or 8 times higher in the APC-DEL group than in the APC-WT or control group (Figure 7B). These factors inhibit the synthesis of viral assembly proteins to prevent virus replication and induce the generation of IFN [18–22]. Altogether, these results reflected a direct anti-viral effect of APC-DEL, which appeared more effective than its indirect antiviral effect manifested as part of its cytoprotective effect.

## DISCUSSION

Mutations in the PROC gene results in PC deficiency, which can be divided into Type I and Type II. Type I deficiency is characterized by an equivalent decrease in protein C antigen and its functional activity. Type II deficiency is manifested as an abnormal functional activity but normal antigen levels [23]. Patients with PC deficiency display different thrombotic tendencies, such as venous thromboembolism and cerebral infarction. Moreover, severe defects may cause neonatal purpura fulminans and disseminated intravascular coagulation (DIC) [5]. Interestingly, some individuals are asymptomatic despite substantial functional abnormality. Diminished anticoagulant activity and cytoprotective effects of certain APC variants can greatly increase the risk of thrombosis, especially when the vascular endothelium is inflamed or mechanically damaged [24]. For instance, the K150del variant is associated with an increased risk of venous thromboembolism and IS due to impaired anticoagulant activity and endothelial barrier protection effect [12–15]. We reported reduced anti-inflammatory of the APC mutant, as demonstrated by enhanced leukocyte recruitment and adhesion, higher endothelial permeability, and altered gene expression profiles. These results corroborate with the previous findings that not only the reduced anticoagulant activity but also impaired cytoprotective effect of the variant promote thrombogenesis [14, 15]. Furthermore, we also demonstrated impaired anti-apoptotic capacity of the K150del variant, which played an important role for nerve repair in IS [7]. Our results comprehensively explained the mechanisms of the APC variant causing IS, provided a supplement for the previous research [13] and could arouse the attention of clinicians for the etiological diagnosis of patients with recurrent IS.

The potent cytoprotective effects of APC, independent of its anticoagulant activity, have been exploited to treat inflammation-related diseases, including sepsis, acute respiratory distress syndrome, ischemic infarction, traumatic encephalopathy, systemic lupus erythematosus, diabetes, and wound healing [24]. Furthermore, APC protects against Ebola and Dengue virus infections by eliminating the infected vascular endothelial cells [25, 26]. We showed that despite the impaired cytoprotective activity of the K150del variant, it exhibited normal protective functions at therapeutic doses. Moreover, compared with the wild APC, the mutant type exerted direct antiviral effects, involving upregulation of the IFN signaling pathway, stimulation of IFN production, inhibition of virus replication, and elimination of infected cells. Normal APC competitively binds to EPCR to downregulate the IFN signaling pathway, whereas the K150del variant promotes IFN production due to a poor affinity for EPCR [27]. Based on these results, we believe that the variant could be used for treating inflammation and viral diseases including 2019 coronavirus disease (COVID-19). Because the pathogenesis of COVID-19 involves a cytokine storm and inflammatory damage to the microvascular system [19, 20, 28], manifested as damaged endothelial barrier, increased vascular permeability, and abnormal activation of coagulation pathways, causing extensive microthromboses, K150del APC could be used as an auxiliary therapy for COVID-19 patients, especially for those with old age, adverse complications, or those intolerant to anti-infective therapies.

To reduce the bleeding risk associated with APC treatment and retain its cytoprotective effects, numerous variants of APC have been developed. For example, 3K3A-APC displayed a good therapeutic effect in clinical trials for cerebral infarction [29]. We found impaired anticoagulant activity of the K150del variant at low concentrations, which returned to normal levels at high doses, indicating the risk of hemorrhage even when the variant is used as a therapeutic drug. This risk can be reduced by appropriately reducing the dose or local administration, such as via lung inhalation and targeted therapy with loaded nanomaterials [30].

A limitation of our study was the inability to construct a coronavirus infection model in mice due to laboratory safety issues. In conclusion, our study provides a comprehensive understanding of the effects of the K150del variant on the functions of APC. In addition, the mutant APC could be used as an auxiliary therapy for COVID-19 patients, especially as a supplement to early treatment for severe patients.

## MATERIALS AND METHODS

### Reagents and animals

Lentiviral vectors were constructed by Hanbio Biotechnology (Shanghai, China). TRIzol reagent was purchased from Takara (Japan). HisTrap excel column was procured from GE (MA, USA). Human Protein C ELISA Kit was purchased from Assaypro (MO, USA) and the HEMOCLOT Protein C kit was provided by Hyphen BioMed (France). EtEraserTMSE endotoxin removal kit and chromogenic end-point tachypleusamebocyte lysate (CETAL) were purchased from Bioendo technology company (Xiamen, China). LPS, thrombin, Evans blue, and peroxidase-conjugated sheep anti-mouse immunoglobulin G (IgG) antibody were obtained from Sigma (MO, USA). Mouse anti-human ICAM-1 monoclonal antibody was purchased from Abcam (England). Mouse anti-human VCAM-1 monoclonal antibody and Vybrant DiD dye were provided by Thermo (MA, USA). Mouse anti-human E-selectin monoclonal antibody and staurosporine were bought from Millipore (MO, USA). Nuclear factor κB (NF-κB) ELISA Kit was purchased from Cell Signaling Technology (MA, USA). Annexin V-fluorescein isothiocyanate (FITC) Apoptosis Detection Kit was provided by Becton, Dickinson and Company (NJ, USA). Other rabbit anti-mouse polyclonal antibodies, including IFIT1, IFITM3, ISG15, OAS1, RSAD2, and GAPDH were purchased from ABclonal (BSN, USA). Enhanced chemiluminescence (ECL) reagent was purchased from Antgene (Wuhan, China). Endothelial cell growth medium (ECM), consisting of 500 mL of basal medium, 25 mL of fetal bovine serum (FBS), 5 mL of endothelial cell growth supplement, and 5 mL of penicillin–streptomycin solution, was purchased from ScienCell (CA, USA). Dulbecco’s modified Eagle’s medium (DMEM), RPMI-1640 medium, phosphate-buffered saline (PBS), FBS, and penicillin–streptomycin solution were purchased from Gibco (CA, USA).

RIPA lysis buffer was provided by Beyotime Biotechnology (Wuhan, China) and protease inhibitor cocktail was purchased from Bimake (TX, USA). Male C57BL/6 mice (6–8 weeks’ old) were provided by Charles River Laboratories (Beijing, China). Animal treatment protocols were approved by the Animal Ethics Committee of Union Hospital, Huazhong University of Science and Technology.

### Cell culture

Human embryonic kidney 293T (HEK 293T) cells expressing wild or mutant PC zymogens were provided by Hanbio Biotechnology (Shanghai, China). These cells were maintained at 37° C and 5% CO_2_ in DMEM supplemented with 10% FBS, 100 IU/mL penicillin, and 100 mg/mL streptomycin [12]. Primary human umbilical vein endothelial cells (HUVECs, a kind gift from B. Hu, originally purchased from ATCC, Manassas, VA, USA) were grown in ECM [16]. Human monocytic leukemia cell line (THP-1), purchased from the cell bank of the Chinese Academy of Sciences, was cultured in RPMI-1640 medium with 10% FBS [31].

### Transcriptome RNA-sequencing (RNA-seq)

Lentiviral particles (Hanbio Biotechnology) were constructed using pHBLV-CMV-wild/mutant PROC-6xHis-GFP vectors and transfected into HEK 293T cells. Stable GFP-positive transformants expressing wild or mutant PC zymogens were screened using flow cytometry. The efficacy of two transfections were normalized to each other according to the fluorescence intensity of GFP under a fluorescence microscope (Olympus, Japan). After allowing the HEK 293T cell lines to grow and adhere to a six-well plate for 12 h, the medium was replaced with serum-free medium containing 10 mg/L vitamin K1 to promote PC secretion [12]. After 48 h, cells were incubated with 1 nM thrombin for 3 h to activate PC derivatives to APC (APC-WT and APC-DEL) [14]. Subsequently, the medium was replaced with serum-free medium and the cells were harvested for 24 h. Finally, cells were collected and the total RNA was extracted using the TRIzol reagent. RNA-Seq was performed by Majorbio Company (Shanghai, China) and results were analyzed using the online software (https://cloud.majorbio.com/).

### Purification of recombinant proteins and detection of anticoagulant activity

Recombinant PC-WT and PC-DEL were collected from 20 L of serum-free conditioned media of HEK 293T cell lines. These were further purified to homogeneity using a HisTrap excel column. PC derivatives (0.5–1.0 mg) were activated to APC by thrombin (25 μg) in TBS buffer (0.1 M NaCl, 0.02 M Tris-HCl, pH 7.4) containing 5 mM ethylenediaminetetraacetic acid for 2 h at 37° C [32]. Excess of thrombin was removed using the HisTrap excel column. The endotoxin removal of APC-WT and APC-DEL was performed using an EtEraserTMSE endotoxin removal kit and the content of endotoxin was detected by CETAL according to protocols. APC samples with endotoxin lower than 0.01 EU/ml were used for cellular and vivo assays later. The purities of APC-WT and APC-DEL were determined by running the samples on a sodium dodecyl sulfate-polyacrylamide gel electrophoresis (SDS-PAGE) gel under non-reducing conditions, followed by staining with Coomassie Blue R-250.

Antigen concentrations of APC-WT and APC-DEL were detected by Human Protein C ELISA Kit and were adjusted to consistent levels (10–100 nM) by PBS. The anticoagulant activity of APC-WT and APC-DEL was measured using a chromogenic substrate with a HEMOCLOT Protein C kit on an automated coagulation analyzer (Diagnostica Stago, France), as described previously [13].

### Endothelial cell permeability assay

To assess the cytoprotective effects of APC, the cell permeability assay was performed using a previously described method [33]. Briefly, HUVECs were plated in the upper chamber of the Transwell system until confluence to a monolayer. They were subsequently incubated with fresh culture medium containing different concentrations of APC for 3 h, followed by LPS treatment (100 ng/mL) for 4 h to induce permeability. After washing with PBS, the medium in the upper chamber was replaced with Evans blue (0.67 mg/mL) diluted in the growth medium containing 4% bovine serum albumin. The medium in the lower chamber was replaced with fresh growth medium. Ten min later, the absorbance of the dye was detected at 650 nm in a 50 μL sample (1:3 dilution) obtained from the lower chamber.

### Expression of cell surface receptors

The expression of cell surface receptors, including ICAM-1, VCAM-1, and E-selectin, was measured using whole-cell ELISA as described previously [34]. Briefly, the cell monolayer of HUVECs was treated with increasing concentrations of APC for 3 h, followed by treatment with LPS (100 ng/mL) for 16 h. The medium was removed, and cells were washed with PBS and fixed with 1% paraformaldehyde for 15 min at room temperature. After washing, cells were incubated with mouse anti-human monoclonal antibodies against ICAM-1, VCAM-1, and E-selectin (1:50) for 1 h (37° C, 5% CO_2_). Next, the cells were washed thrice followed by incubation with sheep anti-mouse IgG antibody (1:2000) for 1 h. The cells were washed again and incubated with 3,3',5,5'-tetramethylbenzidine substrate for 30 min. After adding the stop solution, the absorbance was immediately measured at 450 nm.

### Cell adhesion assay

The adherence of THP-1 cells to endothelial cells was evaluated using a fluorescence-based assay, as described previously. Briefly, THP-1 cells were labeled with Vybrant DiD dye and washed. Next, HUVECs, incubated with APC (10-100 nM, 3 h) followed by incubation with LPS (100 ng/mL, 16 h), were co-cultured with THP-1 cells for 1 h at 37° C (5% CO2). Non-adherent THP-1 cells were gently washed off and the fluorescence of adherent cells was measured. The percentage of adherent THP-1 cells was calculated using the following formula: percentage adherence = (adherent signal/total signal) x 100 [31].

### NF-κB activation

The effect of APC on LPS-stimulated NF-κB activation was estimated using an ELISA kit following the manufacturer’s protocol. In short, HUVECs were incubated with APC (10–100 nM, 3 h) followed by incubation with LPS (100 ng/mL, 16 h). After washing, the nuclear lysates of cells were extracted to estimate the levels of NF-κB [35].

### Anti-apoptotic activity

The cytoprotective effects of APC were determined by inducing apoptosis in HUVECs by staurosporine [17]. Briefly, cells were incubated with APC (5–40 nM) for 5 h before treatment with staurosporine (10 μM, 1 h). Thereafter, viable and apoptotic cells were determined using the Annexin V-FITC apoptosis detection kit following the manufacturer’s protocol.

### Anti-viral effects

To evaluate the anti-viral effects of APC, male mice were divided into three groups (*n* = 4 for each group) and intravenously administered PBS, wild, or mutant APC (5 mg/kg) every 2 days [36]. After 2 weeks, all mice were sacrificed and lung tissues were collected to extract the total cellular protein. Western blotting was performed to assess the levels of IFIT1, IFITM3, ISG15, OAS1, and RSAD2 relative to internal control (GAPDH).

### Western blotting

Total cellular lysates were prepared from lung tissues using RIPA lysis buffer containing 1% protease inhibitor cocktail. Protein samples were separated on 10% SDS-PAGE gels (30 μg per lane). The proteins were transferred onto polyvinylidene difluoride (PVDF) membranes. Afterward, the membranes were blocked with 5% nonfat milk for 1 h and incubated overnight at 4° C with primary antibodies against IFIT1, IFITM3, ISG15, OAS1, RSAD2 and GAPDH (1:1000 for each antibody) followed by incubation with secondary IgG antibody (1:3000) for 1 h. The ECL reagent was used to visualize the target bands; the intensity value of each band was quantified by the Image J software. The expressions of IFIT1, IFITM3, ISG15, OAS1, and RSAD2 relative to GAPDH were determined for all samples.

### Statistical analysis

Data from three independent experiments are presented as mean ± standard deviation (SD). The difference between the groups was analyzed using Student’s *t*-test with GraphPad Prism 8.0 software. The significance level was set at *P* < 0.05.

## References

[1] Foster DC, Yoshitake S, Davie EW. The nucleotide sequence of the gene for human protein C. Proc Natl Acad Sci USA. 1985; 82:4673–77. 10.1073/pnas.82.14.4673 2991887 PMC390448

[2] Esmon CT. The protein C pathway. Chest. 2003 (Suppl 3); 124:26S–32S. 10.1378/chest.124.3_suppl.26S 12970121

[3] Fukudome K, Esmon CT. Identification, cloning, and regulation of a novel endothelial cell protein C/activated protein C receptor. J Biol Chem. 1994; 269:26486–91. 7929370

[4] Mosnier LO, Zlokovic BV, Griffin JH. The cytoprotective protein C pathway. Blood. 2007; 109:3161–72. 10.1182/blood-2006-09-003004 17110453

[5] Dinarvand P, Moser KA. Protein C Deficiency. Arch Pathol Lab Med. 2019; 143:1281–85. 10.5858/arpa.2017-0403-RS 30702334

[6] Bernard GR, Vincent JL, Laterre PF, LaRosa SP, Dhainaut JF, Lopez-Rodriguez A, Steingrub JS, Garber GE, Helterbrand JD, Ely EW, Fisher CJ Jr, and Recombinant human protein C Worldwide Evaluation in Severe Sepsis (PROWESS) study group. Efficacy and safety of recombinant human activated protein C for severe sepsis. N Engl J Med. 2001; 344:699–709. 10.1056/NEJM200103083441001 11236773

[7] Griffin JH, Fernández JA, Liu D, Cheng T, Guo H, Zlokovic BV. Activated protein C and ischemic stroke. Crit Care Med. 2004; 32(5 Suppl):S247–53. 10.1097/01.CCM.0000126127.87484.2B 15118526

[8] Cornet AD, Groeneveld AB, Hofstra JJ, Vlaar AP, Tuinman PR, van Lingen A, Levi M, Girbes AR, Schultz MJ, Beishuizen A. Recombinant human activated protein C in the treatment of acute respiratory distress syndrome: a randomized clinical trial. PLoS One. 2014; 9:e90983. 10.1371/journal.pone.0090983 24632673 PMC3954619

[9] Jackson CJ, Xue M, Thompson P, Davey RA, Whitmont K, Smith S, Buisson-Legendre N, Sztynda T, Furphy LJ, Cooper A, Sambrook P, March L. Activated protein C prevents inflammation yet stimulates angiogenesis to promote cutaneous wound healing. Wound Repair Regen. 2005; 13:284–94. 10.1111/j.1067-1927.2005.00130311.x 15953048

[10] Dhainaut JF, Laterre PF, Janes JM, Bernard GR, Artigas A, Bakker J, Riess H, Basson BR, Charpentier J, Utterback BG, Vincent JL, and Recombinant Human Activated Protein C Worldwide Evaluation in Severe Sepsis (PROWESS) Study Group. Drotrecogin alfa (activated) in the treatment of severe sepsis patients with multiple-organ dysfunction: data from the PROWESS trial. Intensive Care Med. 2003; 29:894–903. 10.1007/s00134-003-1731-1 12712239

[11] Griffin JH, Zlokovic BV, Mosnier LO. Activated protein C, protease activated receptor 1, and neuroprotection. Blood. 2018; 132:159–69. 10.1182/blood-2018-02-769026 29866816 PMC6043978

[12] Tang L, Lu X, Yu JM, Wang QY, Yang R, Guo T, Mei H, Hu Y. PROC c.574_576del polymorphism: a common genetic risk factor for venous thrombosis in the Chinese population. J Thromb Haemost. 2012; 10:2019–26. 10.1111/j.1538-7836.2012.04862.x 22817391

[13] Lu X, Tang L, Xu K, Ma J, Guo T, Mei H, Yang R, Yu J, Wang Q, Yang Y, Jian X, Hu Y. Novel association of a PROC variant with ischemic stroke in a Chinese Han population. Hum Genet. 2013; 132:69–77. 10.1007/s00439-012-1225-8 22976599

[14] Ding Q, Yang L, Hassanian SM, Rezaie AR. Expression and functional characterisation of natural R147W and K150del variants of protein C in the Chinese population. Thromb Haemost. 2013; 109:614–24. 10.1160/TH12-10-0760 23389250 PMC3634890

[15] Yamashita A, Zhang Y, Sanner MF, Griffin JH, Mosnier LO. C-terminal residues of activated protein C light chain contribute to its anticoagulant and cytoprotective activities. J Thromb Haemost. 2020; 18:1027–38. 10.1111/jth.14756 32017367 PMC7380734

[16] Bae JS, Rezaie AR. Activated protein C inhibits high mobility group box 1 signaling in endothelial cells. Blood. 2011; 118:3952–59. 10.1182/blood-2011-06-360701 21849480 PMC3193270

[17] Mosnier LO, Griffin JH. Inhibition of staurosporine-induced apoptosis of endothelial cells by activated protein C requires protease-activated receptor-1 and endothelial cell protein C receptor. Biochem J. 2003; 373:65–70. 10.1042/bj20030341 12683950 PMC1223481

[18] Abbas YM, Laudenbach BT, Martínez-Montero S, Cencic R, Habjan M, Pichlmair A, Damha MJ, Pelletier J, Nagar B. Structure of human IFIT1 with capped RNA reveals adaptable mRNA binding and mechanisms for sensing N1 and N2 ribose 2'-O methylations. Proc Natl Acad Sci USA. 2017; 114:E2106–15. 10.1073/pnas.1612444114 28251928 PMC5358387

[19] Londrigan SL, Wakim LM, Smith J, Haverkate AJ, Brooks AG, Reading PC. IFITM3 and type I interferons are important for the control of influenza A virus replication in murine macrophages. Virology. 2020; 540:17–22. 10.1016/j.virol.2019.11.003 31731106

[20] Perng YC, Lenschow DJ. ISG15 in antiviral immunity and beyond. Nat Rev Microbiol. 2018; 16:423–39. 10.1038/s41579-018-0020-5 29769653 PMC7097117

[21] Kurokawa C, Iankov ID, Galanis E. A key anti-viral protein, RSAD2/VIPERIN, restricts the release of measles virus from infected cells. Virus Res. 2019; 263:145–50. 10.1016/j.virusres.2019.01.014 30684519 PMC6615567

[22] Lee WB, Choi WY, Lee DH, Shim H, Kim-Ha J, Kim YJ. OAS1 and OAS3 negatively regulate the expression of chemokines and interferon-responsive genes in human macrophages. BMB Rep. 2019; 52:133–38. 10.5483/BMBRep.2019.52.2.129 30078389 PMC6443328

[23] Wypasek E, Undas A. Protein C and protein S deficiency - practical diagnostic issues. Adv Clin Exp Med. 2013; 22:459–67. 23986205

[24] Griffin JH, Zlokovic BV, Mosnier LO. Activated protein C: biased for translation. Blood. 2015; 125:2898–907. 10.1182/blood-2015-02-355974 25824691 PMC4424414

[25] Hensley LE, Stevens EL, Yan SB, Geisbert JB, Macias WL, Larsen T, Daddario-DiCaprio KM, Cassell GH, Jahrling PB, Geisbert TW. Recombinant human activated protein C for the postexposure treatment of Ebola hemorrhagic fever. J Infect Dis. 2007 (Suppl 2); 196:S390–99. 10.1086/520598 17940975

[26] Cabello-Gutiérrez C, Manjarrez-Zavala ME, Huerta-Zepeda A, Cime-Castillo J, Monroy-Martínez V, Correa BB, Ruiz-Ordaz BH. Modification of the cytoprotective protein C pathway during Dengue virus infection of human endothelial vascular cells. Thromb Haemost. 2009; 101:916–28. 19404546

[27] Liang HP, Kerschen EJ, Hernandez I, Basu S, Zogg M, Botros F, Jia S, Hessner MJ, Griffin JH, Ruf W, Weiler H. EPCR-dependent PAR2 activation by the blood coagulation initiation complex regulates LPS-triggered interferon responses in mice. Blood. 2015; 125:2845–54. 10.1182/blood-2014-11-610717 25733582 PMC4424632

[28] Huang C, Wang Y, Li X, Ren L, Zhao J, Hu Y, Zhang L, Fan G, Xu J, Gu X, Cheng Z, Yu T, Xia J, et al. Clinical features of patients infected with 2019 novel coronavirus in Wuhan, China. Lancet. 2020; 395:497–506. 10.1016/S0140-6736(20)30183-5 31986264 PMC7159299

[29] Lyden P, Pryor KE, Coffey CS, Cudkowicz M, Conwit R, Jadhav A, Sawyer RN Jr, Claassen J, Adeoye O, Song S, Hannon P, Rost NS, Hinduja A, et al, and NeuroNEXT Clinical Trials Network NN104 Investigators. Final Results of the RHAPSODY Trial: A Multi-Center, Phase 2 Trial Using a Continual Reassessment Method to Determine the Safety and Tolerability of 3K3A-APC, A Recombinant Variant of Human Activated Protein C, in Combination with Tissue Plasminogen Activator, Mechanical Thrombectomy or both in Moderate to Severe Acute Ischemic Stroke. Ann Neurol. 2019; 85:125–36. 10.1002/ana.25383 30450637 PMC6342508

[30] Bo L, Bian J, Li J, Wan X, Zhu K, Deng X. Activated protein C inhalation: a novel therapeutic strategy for acute lung injury. Med Sci Monit. 2011; 17:HY11–13. 10.12659/MSM.881789 21629195 PMC3539554

[31] Bae JS, Lee W, Rezaie AR. Polyphosphate elicits pro-inflammatory responses that are counteracted by activated protein C in both cellular and animal models. J Thromb Haemost. 2012; 10:1145–51. 10.1111/j.1538-7836.2012.04671.x 22372856 PMC3366017

[32] Ding Q, Yang L, Dinarvand P, Wang X, Rezaie AR. Protein C Thr315Ala variant results in gain of function but manifests as type II deficiency in diagnostic assays. Blood. 2015; 125:2428–34. 10.1182/blood-2014-12-617274 25651845 PMC4392011

[33] Feistritzer C, Riewald M. Endothelial barrier protection by activated protein C through PAR1-dependent sphingosine 1-phosphate receptor-1 crossactivation. Blood. 2005; 105:3178–84. 10.1182/blood-2004-10-3985 15626732

[34] Bae JS, Yang L, Manithody C, Rezaie AR. Engineering a disulfide bond to stabilize the calcium-binding loop of activated protein C eliminates its anticoagulant but not its protective signaling properties. J Biol Chem. 2007; 282:9251–59. 10.1074/jbc.M610547200 17255099

[35] Dinarvand P, Hassanian SM, Qureshi SH, Manithody C, Eissenberg JC, Yang L, Rezaie AR. Polyphosphate amplifies proinflammatory responses of nuclear proteins through interaction with receptor for advanced glycation end products and P2Y1 purinergic receptor. Blood. 2014; 123:935–45. 10.1182/blood-2013-09-529602 24255918 PMC3916882

[36] Lichtnekert J, Rupanagudi KV, Kulkarni OP, Darisipudi MN, Allam R, Anders HJ. Activated protein C attenuates systemic lupus erythematosus and lupus nephritis in MRL-Fas(lpr) mice. J Immunol. 2011; 187:3413–21. 10.4049/jimmunol.1101125 21849682

