# Correction for: Supplementary research on K150del variant of activated protein C

**DOI:** 10.18632/aging.203437

**Published:** 2021-08-13

**Authors:** Wen-Yi Lin, Liang Tang, Xuan Lu, Yu Hu

**Affiliations:** 1 Institute of Hematology, Union Hospital Affiliated to Tongji Medical College, Huazhong University of Science and Technology, Wuhan, China

**Keywords:** correction

Original article: Aging. 2021; 13:12466–12478.  . https://doi.org/10.18632/aging.202904

**This article has been corrected:** The authors requested to modify the affiliation. The authors declare that these correction does not change the results or conclusions of this work.

The corrected affiliation is posted below.

Institute of Hematology, Union Hospital, Tongji Medical College, Huazhong University of Science and Technology, Wuhan, China

